# Comparative Analysis of Physical Activity, Performance-Related Health, and Academic Achievements in 11-to-13-Year-Old Schoolchildren in Qatar

**DOI:** 10.3390/healthcare12050588

**Published:** 2024-03-04

**Authors:** Souhail Hermassi, Sascha Ketelhut, Ferman Konukman, Maha Sellami, Senaid Al-Marri, Claudio R. Nigg, René Schwesig

**Affiliations:** 1 Physical Education Department, College of Education, Qatar University, Doha 2713, Qatar; fkonukman@qu.edu.qa (F.K.); msellami@qu.edu.qa (M.S.); sealmarri@qu.edu.qa (S.A.-M.); 2 Institute of Sport Science, University of Bern, 3012 Bern, Switzerland; sascha.ketelhut@unibe.ch (S.K.); claudio.nigg@unibe.ch (C.R.N.); 3 Department of Orthopedic and Trauma Surgery, Martin-Luther-University Halle-Wittenberg, 06120 Halle (Saale), Germany; rene.schwesig@uk-halle.de

**Keywords:** body mass index, anthropometrics, overweight, obesity, academic performance

## Abstract

Age-related differences in physical activity (PA), maturity status (PHV), physical performance (PP), and academic achievement (AA) among schoolchildren in Qatar were examined. Sixty-nine students from a school in Doha were categorized into three equal (n = 23) groups: 11-year-old students (U11; male: n = 14), 12-year-old students (U12: male: n = 7), and 13-year-old students (U13: male: n = 11). The testing process comprised a medicine ball throw, Stork balance test, hand grip strength test, the T-half test (PP), GPA in Arabic, mathematics, science (AA), International Physical Activity Questionnaire Short Form (PA), and Moore’s equations (PHV). Relevant age-related differences (*p* < 0.001) were identified in mathematics, science, the T-half test, maturity, and arm span. Notably, differences between adjacent age groups were evident between U11 and U12, concerning arm span, maturity, mathematics, and science, and between U12 and U13 (the T-half test, mathematics, science). Concerning AP, the performance maxima were calculated for U12 (mathematics, science) and U11 (Arabic). Regarding PP, performance maxima were only observed for U13. Except for the moderate level, the highest levels of PA were detected in U13. Maturity status and anthropometric parameters did not differ significantly between age groups. However, AA demonstrated the most notable age-related differences. Specifically, mathematics showed substantial differences between adjacent age groups.

## 1. Introduction

Individuals go through physiological changes as they enter adolescence, such as changes in body fat, body mass index (BMI), and PP, which have an impact on how much PA is performed [1]. When PA is controlled for biological age, the timing of these changes in groups of the same chronological age can have an impact on the tracking of PA [1,2]. In order to determine if PA tracking has improved, investigations evaluate the stability of PA from childhood to late adolescence while correcting for variations in biological maturation [3].

Maturity is the process that characterizes human growth and development, with temporal individual variation [4,5]. In the process of biological growth, it takes approximately 20 years for a newborn to reach biological maturity through the physiological, anthropometric, psychological, and physical development process [4,5]. For example, the magnitudes of muscle power and strength development promptly increase during the progress of maturity until the age of 20 years [6]. Consequently, growth and maturation levels are crucial in terms of evaluating youth physical and technical competencies [7,8].

PA is defined as any body movement that leads to a significant increase in energy expenditure above baseline [9]. PA can be seen in a variety of ways and is heavily influenced by cultural traditions. The frequency, duration, and intensity of total PA are typically used to calculate it.

Despite the well-established benefits of PA on health and wellbeing [10], PA levels have decreased worldwide in recent years, which has been attributed to factors such as the increased use of entertainment technology [11]. Global estimates show that less than 20% of children reach the recommended 60 min of moderate-to-vigorous physical activity per day [12], and it is concerning that this percentage is gradually decreasing [13]. PA has wide-ranging benefits for children’s physical and mental health [14]. Conversely, sedentary behavior (SB) causes negative health consequences [14]. Physical activity is often emphasized by advocates as a way to enhance academic achievement. Hermassi et al. [15] suggest that frequent PA sessions should be incorporated during the school day to improve attention and learning, according to proponents. Increasing PA levels and limiting SB can have several health advantages for children and teenagers. Regularly engaging in high levels of PA and reducing SB is associated with a range of cognitive benefits, positive physical outcomes, and favorable mental health outcomes [16,17].

Adolescents seem to experience a decrease in physical activity that is more closely linked to their biological age than their chronological age [18,19]. The pattern of PA in children and adolescents may change due to biological maturation. Timing and time are two factors that can be used to study biological maturation, which is the journey towards the state of maturity. An event’s “timing” is defined as the time at which it takes place.

Regardless of chronological age, individuals grow less physically active as they approach the condition of maturity [20]. This decline in PA may be attributed to the variable timing of sexual maturation and the growth spurt related to age and gender [20]. Girls’ perceptions of discomfort and poorer self-esteem brought on by the development of secondary sexual features may encourage a decline in PA engagement [21]. Increased body fat and hormonal changes, which are typical of this phase, may be linked to reduced PA [22]. Regarding early peak height velocity (PHV) in boys, the age at which this occurs can positively influence the behavior of PA due to increased strength and muscle mass, which tends to occur after the PHV point [23].

Children who possess superior physical performance also achieve more academic success [15]. Various studies have reported a positive association between academia and PP [15,24], which justifies school-based physical performance programs which aim to develop and improve physical performance. In addition to the transferrable skills of physical performance that can positively influence academic performance, it has also been associated with improved cognitive ability (probably permitting greater academic performance).

As a result, this improved cognitive ability can also have a direct and positive impact on specific sports performance, serving dual purposes [24]. During growth, data have consistently supported the efficacy of PA [25,26]. Before we can comprehend the causal relationship between growth, maturity, elevated PA, and academic performance related to cognitive behavior—including the significance of particular age group characteristics—we still need to learn more. It is probable that interventions could be improved further by means other than just extending their duration.

In brief, the paucity of research concerning maturity status, PA, PP, health-related components, and academic achievements is even more pronounced among students in different age groups. 

It is clear that PA decreases with age as children progress from childhood to adolescence [27], but previous studies have drawn inconsistent conclusions about the patterns of these changes across demographic groups [28,29,30]. For example, while studies have consistently reported that PA levels are higher in boys than girls, the findings regarding patterns of age-related change in the two genders have not been as clear. Farooq et al. [31] have concluded that a decline in physical activity begins at an earlier age in girls than boys, whereas Reilly et al. [32] have suggested that this decline begins at the age of entry to gendered schools and follows similar trajectories in both sexes. 

These inconsistencies should be resolved so that PA can be focused on reducing age-related decreases in PA in demographic sub-groups, in which the decline is most pronounced. Further, such interventions should be designed with a knowledge of the critical age ranges during which physical activity decreases most rapidly. Therefore, data concerning countries from the Gulf region are scarce, and this geographical area has unique features, which means that research from other parts of the world may not be applicable to the Gulf. However, such information is vital for policy formulation, intervention design, and implementation among children in physical education classes [15,33]. There have been no previous studies that have examined the maturity status, PA, health-related components, and academic achievements in different ages. Furthermore, we believe it is important to collect reference data about schoolchildren for future reference. As a pathway to enhance children’s health and physical performance, such profiles could provide benefits for long-term athletic development. 

The main objective was to elaborate age-dependent differences concerning several aspects. In detail, the aims of this study were to analyze the differences regarding different dimensions: maturity status, PP, PA, and AA among 11-to-13-year-old schoolchildren in Qatar. We hypothesized a priori that PP, PA, and AA would be different between students of different ages.

## 2. Materials and Methods

### 2.1. Research Protocol

This study employed a cross-sectional design, recruiting healthy schoolchildren from an urban school within the Doha community (Qatar) using convenience sampling. All assessments were conducted over four consecutive days following a predetermined sequence. Anthropometric measurements were taken on the first day. On the second day, the Stork balance test was performed. The third day encompassed the medicine ball throw test and completion of the PA questionnaire. Two weeks following the first testing period, the assessments conducted on days 1 to 3 were repeated to evaluate test–retest reliability. The values from the second test session were utilized for analysis. 

All assessments were conducted indoors within a consistent time frame (from 8:00 to 10:00 a.m.) and under controlled environmental conditions (temperature 24.5 ± 0.5 °C; relative humidity 65 ± 5%). Throughout the testing period, participants adhered to their usual self-reported dietary intake of food and fluids. Additionally, participants were instructed to abstain from engaging in vigorous PA, consuming caffeine-containing beverages, or eating anything within 24 h, 4 h, and 2 h preceding the testing, respectively.

On the second and third test days, a standardized warm-up protocol was conducted. The warm-up began with a general phase involving five minutes of low-intensity running. Subsequently, participants performed three 15 m acceleration sprints, and a maximum 20 m sprint, with each sprint separated by three minutes of passive recovery. Additionally, various submaximal dynamic stretches and throwing exercises were performed.

### 2.2. Participants

Prior to any data collection, the administrative team, school authorities, participating children, and their parents were informed of the study objectives, risks, and procedures. Participants, along with their respective guardians, were informed about their right to withdraw from the experiment at any time and they provided written informed consent. Sixty-nine healthy schoolchildren (body mass: 53.8 ± 14.9 kg; height: 1.57 ± 0.08 m; BMI: 21.6 ± 5.10 kg/m^2^; seat height: 120 ± 5.76 cm; arm span: 156 ± 9.63 cm) were included in the final analyses.

### 2.3. Inclusion and Exclusion Criteria

Exclusion criteria: The inclusion criteria specified that all the children must be healthy and able to exercise. If a participant fitted any of the following, they were excluded: (1) a psychiatric condition; (2) taking medication (such as antidepressants or drugs that impact the nervous system); and (3) did not provide a completed informed permission form.

Inclusion criteria: Participants were eligible to participate if they (1) provided written informed parental or guardian consent, (2) were in a good health, and had no contraindications for physical activity, (3) had no physical limitations to exercise, and (4) were in the age range of 10–13 years.

### 2.4. Ethical Approval

This study was approved by the Ministry of Education and Higher Education of Qatar (REF: 18/2021) and the ethical institutional review board of Qatar University (QU-IRB 1544-FBA/21—Date of approval on 18 June 2023) and was con-ducted in accordance with the Declaration of Helsinki. Formal written consent was obtained from the parents/guardians of the participants. All the children submitted an informed consent form signed by their parent/guardian, giving verbal assent before being assessed. The children’s participation was voluntary, and it was explained that withdrawal from the study was possible at any point without further obligation.

### 2.5. Procedures and Assessements

#### 2.5.1. Anthropometry

Body mass (model TBF 105; Tanita Corporation of America, Inc., Arlington Heights, IL, USA) and height (Holtain stadiometer, Crosswell, Crymych, Pembrokeshire, UK) were measured to the nearest 0.1 kg and 0.1 cm, respectively. BMI was calculated by dividing body mass by the square of height (kg/m^2^). Body fat was measured to the nearest 0.1 mm using Harpenden calipers (Baty International, Burgess Hill, Sussex, UK).

#### 2.5.2. Biological Maturity

To predict participants’ maturity offset, gender-specific equations were utilized: Boys: Maturity offset (years) = −8.128741 + (0.0070346 (age sitting height)). Girls: Maturity offset (years) = −7.709133 + (0.0042232 (age stature)) [34].

#### 2.5.3. Physical Performance

Postural control was assessed utilizing the Stork balance test [35]. Medicine ball throws [36] were performed using a 3 kg medicine ball with a diameter of 21.5 cm. The T-half test [37] was conducted to determine agility. The hand grip strength of the dominant hand was measured using a digital hand grip dynamometer (T.K.K. 5401, Tokyo, Japan) with a sensitivity of 10 N. The PP tests used in this study have been previously described in detail [15,38].

#### 2.5.4. Academic Achievement

Retrospective analysis of the children’s grade point average (GPA) and participants’ subject-specific percentage in Arabic language, mathematics, and science in the academic year 2021–2022 were used to determine AA. The reason for including mathematics and science courses was due to our interest in science courses [39].

#### 2.5.5. Physical Activity Questionnaire

Self-reported PA was assessed using the IPAQ-SF, a valid and reliable tool for assessing PA across diverse populations [40]. The Arabic version of the IPAQ-SF was utilized in this study [41]. The questionnaire enabled the calculation of minutes per day spent in moderate to vigorous PA. Following the IPAQ-SF scoring protocol [42], PA was classified into three intensity levels: moderate (4 METs), vigorous (8 METs), and walking (3.3 METs) [43]. Additionally, total PA, which encompassed the sum of these three intensities, was computed.

### 2.6. Statistical Analysis

Prior to inference statistical analyses, all variables were tested for normal distribution (Shapiro–Wilk Test) and the assumption of variance homogeneity (Levene test for equality of variances). Descriptive statistics [mean, standard deviation (SD), minimum, maximum, and 95% confidence intervals (95% CI)] were ascertained for all variables. Differences in anthropometric parameters, AA, and PA parameters between age groups were tested using the Kruskal–Wallis H-test and Mann–Whitney U test for post hoc testing, as the data were not normally distributed. The threshold for statistical significance and relevance was set at α < 0.05 and η_p_^2^ > 0.15 [44,45].

A power calculation (nQuery Advisor 4.0; Statistical Solutions, Saugus, MA, USA) was performed using previous data [15]. Based on the main parameter, science, a *t*-test for independent groups, a mean difference of 6.5 (pooled SD: 6.9; d = 0.91), a significance level of 5% and a power of 80%, and a sample size of 20 participants in each group are necessary [46].

Pearson’s product–moment correlations were calculated to determine the relationship between anthropometric parameters, AA, PA, and PP parameters. The following criteria were adopted for interpreting the magnitude of correlation (r) between the measures: <0.1, trivial; 0.1–0.3, small; 0.3–0.5, moderate; 0.5–0.7, large; 0.7–0.9, very large; or 0.9–1.0, almost perfect [46]. The relationships between the variables were analyzed with Spearman’s product–moment correlation (r) and interpreted as negligible (<0.1), weak (0.1–0.4), moderate (0.4–0.7), strong (0.7–0.9), or very strong (>0.9). A correlation coefficient of at least r^2^ > 0.5 was considered relevant. Regarding the sample size of n = 53, the critical value for the product–moment correlation based on a two-sided *t*-test and a = 5% is r = 0.270 [47]. 

Statistical analysis was performed using SPSS version 28.0 for Windows (SPSS Inc., IBM, Armonk, NY, USA).

## 3. Results

### 3.1. Normal Distribution and Varinace Homogeneity

Except for body height and arm span, all variables exhibited non-normal distributions. Consequently, median and arithmetic means are presented for comparison with previous studies. Nonparametric tests evaluated the differences between age groups. Five parameters (seat height: *p* = 0.045; postural control: *p* = 0.002; mathematics: *p* = 0.042, science: *p* = 0.006, walking MET-minutes/week: *p* = 0.006) were heterogeneous in variance.

### 3.2. Anthropometric Data

Except for maturity status (*p* < 0.001) and arm span (*p* < 0.001), anthropometric parameters were not different between the age groups (Table 1 and Table 2). The largest differences between the adjacent age groups were found between U11 and U12 (*p* < 0.001; Table 2). All maxima were calculated for either U12 (body height, seat height, maturity status), or U13 (body weight, arm span, BMI).

### 3.3. Physical Performance

The hand grip strength (*p* = 0.033) and agility T-half tests (*p* < 0.001) showed relevant differences between age groups (Table 3). For agility the T-half test, the differences between U12 and U13 was significant (*p* < 0.001). Apart from postural control (U12), the U13 showed the highest performance level in all parameters.

### 3.4. Academic Performance

**Figure 1:**
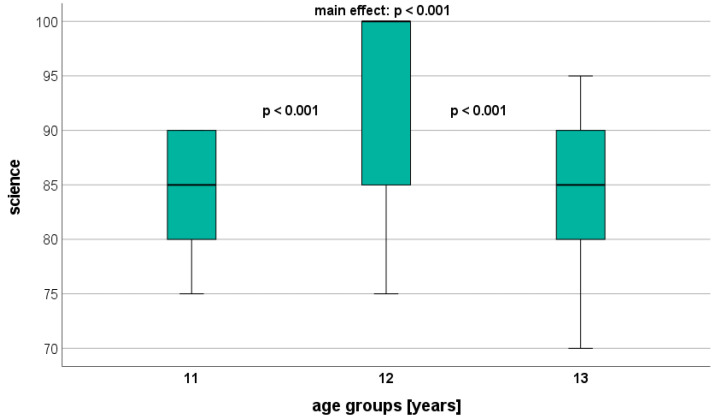
Performance level in science (GPA) depending on age.

**Figure 2:**
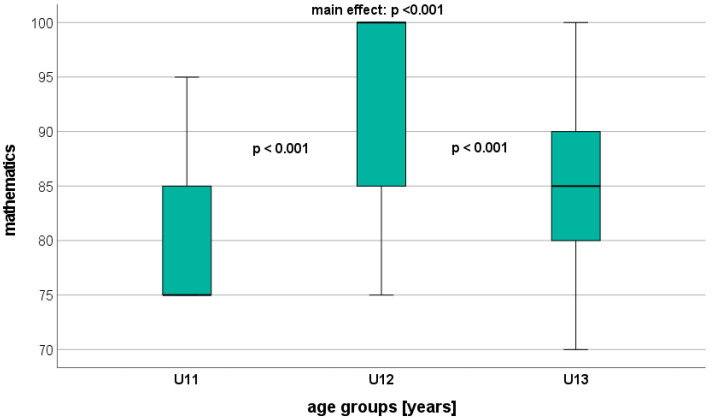
Performance level in mathematics (GPA) depending on age.

**Figure 3:**
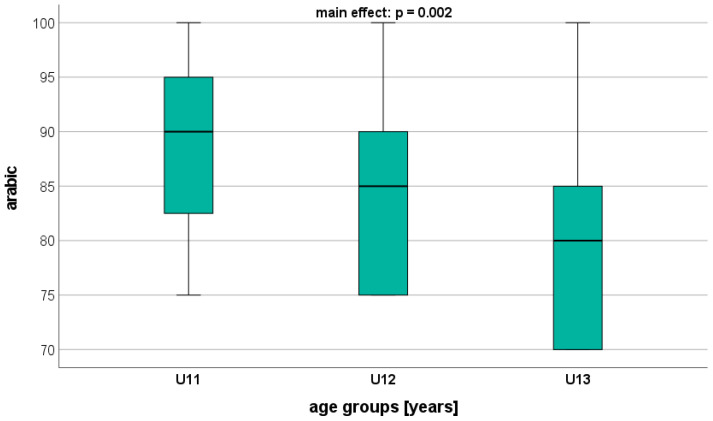
Performance level in Arabic (GPA) depending on age.

Overall, significant age-related differences were found in all AA parameters, with AA showing the largest differences (Table 4). Science and mathematics, in particular, dis-played significant differences between the adjacent age groups (U11 vs. U12: *p* < 0.001; U12 vs. U13: *p* < 0.001) (Figure 1 and Figure 2). Regarding Arabic, a significant difference was observed only between U11 and U13 (*p* = 0.002) (Figure 3). On a descriptive level, the highest AA was identified in U12 for mathematics and science, and in U11 for Arabic.

### 3.5. Physcal Activity

None of the PA parameters exhibited a significant difference between age groups (Table 5). The highest MET-minutes/week were calculated for U11 (moderate) and U13 (vigorous, walking, total). No significant correlations (r > 0.5) were observed among the different parameters (anthropometric, PP, AA, PA).

## 4. Discussion

This study investigated the age-dependent differences in PA, maturity status, PP, and AA in 11-to-13-year-old students in Qatar for a better understanding of the interaction between the several mentioned dimensions.

Significant differences were noted in maturity status and arm span across age groups. The hand grip strength and agility T-half test were the PP parameters that exhibited the most substantial differences between age groups. AA exhibited significant age-related differences across all three parameters. Particularly, mathematics showed large differences between adjacent age groups. However, no significant differences were found in any of the PA parameters across different age groups.

### 4.1. Maturity Status

According to the present results, the most substantial differences in maturity status in our study were detected between U11 and U12. This is in line with previous research showing that older children generally exhibit a higher level of maturity compared to their younger peers, potentially resulting in initially superior performance [48]. 

Previous research suggests that children’s participation in PA may be influenced by their maturity status, as it affects both psychosocial and biological elements, which are likely to have an impact on PA levels [49].

According to reports, the differences in MVPA between early-, average-, and late-maturing boys and girls could mostly be explained by differences in chronological age. To have a comprehensive understanding of the effects of early, average, or late maturation on physical activity levels in adolescence, more research is needed in this field.

### 4.2. Physical Activity

Regular engagement in moderate-to-vigorous PA significantly contributes to individuals’ well-being and health by reducing the prevalence of overweight and obesity [50], decreasing levels of anxiety and depression, and lowering the general risk of non-communicable diseases [51]. According to the present results, none of the PA parameters showed any significant difference between age groups. The highest energy expenditures depending on intensity level (vigorous vs. moderate vs. walking) were calculated for U11 (moderate) and U13 (vigorous, walking, and total). The different PA levels (vigorous vs. moderate vs. walking) did not show any relevant (r < 0.5) correlation regarding the maturity status.

The gender differences among participants in our study on PA behavior are still debated, and it is important to take note of this; according to some studies, boys are found to engage in more moderate-to-vigorous physical activity (MVPA) than girls [52,53], while others indicate that there are no differences [54]. In childhood and adolescence, PA tends to decline with age, according to earlier studies [55,56,57]. Many of those studies, however, employed cross-sectional designs, which might have resulted in findings that do not accurately reflect patterns that would be obvious with prospective, longitudinal study designs [28,58]. Additionally, only a few of the earlier longitudinal studies of PA have taken into account the likelihood that certain sub-groups of children may exhibit various patterns of change as they enter adolescence [28].

### 4.3. Physical Performance

In our study, hand grip strength performance significantly differed between age groups. Our findings show that hand grip strength gradually rises linearly with age. Studies published in the past from other countries have provided support for these conclusions [59,60]. We showed a correlation between age and hand grip strength. According to the findings of this experiment, grip strength significantly increased between the ages of 10 and 12 years old, which is consistent with the findings of Hager-Ross and Rosblad [61]. This apparently concurs with physical growth associated with the onset of puberty. In addition, biological maturity is known to have a strong impact on strength measures, especially for boys [62,63].

Numerous research studies on hand grip strength from various populations and age groups have been published [64]. There are only a few studies that have been published on the average hand grip strength of children [59,60]. The most recent studies have provided data from children in Sweden [61] and Korea [59]. Earlier studies were from children in the USA [64] and Australia [65]. The results from these studies have shown that grip strength may vary across age groups. Moreover, Jenue et al. [66] have concluded that hand grip strength may differ across regions. 

The use of agility tests that combine measures of change of direction and speed is encouraged in school practices. Since agility skills represent a crucial part of the performance in many sports, their assessment should be considered an integral part of functional testing in schoolchildren [67]. In the current study, the agility T-half test was the PP parameter exhibiting the largest differences between age groups. In addition, the agility performances between U12 and U13 was significantly different. This result is consistent with that of other authors [68] who have examined the agility reaction time in young people. These findings support the theory that differences in processing speed with age are due to a generic (i.e., non-task-specific) component that evolves quickly throughout childhood and more slowly during adolescence.

However, Zemková et al. [69] have reported that schoolchildren’s agility time dropped very dramatically between the ages of 7 and 10 (27.1%) and 10 and 14 (26.5%). As a result, it is possible to draw the conclusion that agility time decreases with age up to early maturity. Since this is one of the first studies to use the reactive agility test to evaluate agility abilities, the results could be used for comparing people of similar ages. A common pattern is a decline in agility mean and variability in childhood and adolescence, followed by an increase in mean and variability in adulthood and old age, which was also confirmed in a study by Dykiert et al. [70]. 

To conclude, this observation could be a result of either peak physical maturation rates or limited lower body-specific skills in these age groups. This is validated by the observation that pre-adolescent children with advanced biological maturity exhibit greater PP (e.g., in speed, strength, and power) than those who mature later [71,72].

No significant age-related difference in the medicine ball throw was found in this study. These results contradict Hermassi et al. [73], who reported average differences in MBT between groups of school-aged athletes of 6% (U10 to U11) and 15% (U11 to U12). These results are further supported by a recent study [38], also reporting a significant difference between U11 and U12 school-aged soccer players in MBT. These findings offer data for the evaluation and assessment of player performance and might be used to construct and improve positional training regimens in school PE programs. The prevention, assessment, and treatment of injuries frequently sustained by schoolchildren may also benefit from these findings.

A child’s development is greatly impacted by their ability to maintain postural control, which is a necessary condition for performing complex motor skills and skilled movements with competence. This study revealed no discernible age-related variations in postural stability. Numerous investigations evaluated children’s balance, and the findings indicated that children aged 7 to 9 have different motor skills when they are performing cognitive and motor tasks simultaneously [74,75]. Ghanbarzadeh et al. [76] have shown that postural control improved with advancing age in all tasks. In simple tasks, 9-year-old children’s postural sway was the same as that of adults. However, when the task demands were higher, a significant difference in postural sway was observed between children aged seven to twelve. As a result, until the age of twelve, postural stability has not developed. Therefore, identifying children who are more likely to fall and developing fall prevention strategies depend on a better understanding of postural control.

### 4.4. Academic Performance

Globally, research on how age affects school-age children’s cognitive, motor, and emotional skill development has accelerated in recent decades [77]. This is partly because public policies that aim to enhance the teaching and learning processes that are part of every educational system have been implemented and revised. According to this study’s findings, substantially older students in a class do significantly better on academic achievement exams than their comparably younger colleagues [78,79]. In addition, Hermassi et al. [15] have found significant group differences in academic performance parameters, such as mathematics and science, between obese and non-obese groups of schoolchildren aged 13 years. For Arabic language, the group difference did not reach the level of significance.

An age impact for science was found to be significant in a recent study that looked at academic performance in school-aged athletes [38]. The performance difference between U10 and U11 is the basis for the primary age effect. Numerous studies have examined the degree to which academic achievement predicts later-life body mass, and many of these studies also concentrate on the reverse causal link, which examines how body mass impacts educational achievements [15,33,41].

The age gap in academic performance is significantly smaller for schoolchildren of the same relative age but with an adequate family climate of support and involvement in their education than it is for those with an inadequate family climate [80,81]. According to several recent studies, children who start school later than their peers have a number of short- and medium-term benefits, including higher test scores throughout primary and secondary school, a higher development of non-cognitive skills, and a lower likelihood of committing crimes [77,81,82]. 

### 4.5. Limitations

This study has some limitations, such as the controls for other potential confounders, which were not measured (e.g., school environment, social interaction, family situation, children’s ethnicity, school stress). Such psychological aspects should be more intensively investigated in future studies. Another drawback is that since this study was cross-sectional, causality cannot be assigned. Additionally, the outcomes might have been different if the study had included more people. The results should not be generalized without caution. For example, the relation between males and females was markedly different in the three age groups. This could explain the atypical age-dependent academic performance with performance maxima in the U12 (mathematics, science) and U11 (Arabic) groups.

In summary, an analysis of other studies suggests that more significant differences between age groups are found when larger sample sizes are studied or when the duration of the research is longer [83,84].

## 5. Conclusions

Significant differences were noted in maturity status among age groups, and the hand grip strength and agility T-half test were the PP parameters that exhibited the most substantial differences between age groups. AA exhibited significant age-related differences particularly, and mathematics showed large differences between adjacent age groups. However, the highest MET-minutes/week values were calculated for the U11 (moderate) and U13 (vigorous, walking, total) groups. However, this study offers insightful information that can be used to assess students’ physical and intellectual performance. Particularly significant factors include the age dependence, the interplay of many variables (anthropometric, physical, and academic performance), and the longitudinal growth over a period of three years. These results therefore have implications for scientists, trainers, and instructors of physical education who work with children, especially regarding the different treatment of males and females in this age range.

Future research in this field is necessary for a deeper understanding of the relative significance of biological and behavioral variables in the well-documented reduction in PA levels during adolescence. Therefore, further studies at these ages with different samples would be interesting to pursue. Additionally, study should be conducted to ascertain whether the link between physical activity and maturity is causative or may be explained by other variables that may affect development throughout the peripubertal era. Future studies on this topic should assess technical skills during physical education classes to identify any potential age differences.

## Figures and Tables

**Table 1:** Basic anthropometric characteristics for the different age groups. Maxima marked in bold.

	Body Height (m)	Body Mass (kg)	BMI (kg/m^2^)
	Median Mean ± SD (95% CI)	Median Mean ± SD (95% CI)	Median Mean ± SD (95% CI)
U11 (n = 23)	1.52 1.53 ± 0.08 (1.51–1.59)	46.0 51.0 ± 13.8 (44.8–57.3)	19.9 21.7 ± 5.63 (19.5–23.8)
U12 (n = 23)	1.59 1.59 ± 0.08 (1.56–1.63)	55.0 53.7 ± 12.6 (47.4–59.9)	21.8 21.1 ± 4.14 (18.9–23.2)
U13 (n = 23)	1.58 **1.59 ± 0.08** (1.55–1.64)	50.0 **56.7 ± 18.0** (50.4–62.9)	20.9 **22.1 ± 5.57** (20.0–24.3)
Kruskal–Wallis (*p*)	***p* = 0.023**	*p* = 0.449	*p* = 0.792
Significant differences between adjacent age groups (*p*)	**U11/U12:** ***p* = 0.044**		

**Table 2:** Special anthropometric characteristics for the different age groups. Maxima marked in bold.

	Seat Height (cm)	Arm Span (cm)	Maturity Status
	Median Mean ± SD (95% CI)	Median Mean ± SD (95% CI)	Median Mean ± SD (95% CI)
U11 (n = 23)	119 118 ± 4.52 (116–120)	149 148 ± 7.34 (145–152)	−1.72 −1.46 ± 0.82 (−1.79–−1.13)
U12 (n = 23)	121 **121 ± 3.40** (119–124)	158 159 ± 7.46 (155–162)	0.04 **−0.10 ± 0.74** (−0.43–0.23)
U13 (n = 23)	122 121 ± 7.99 (119–123)	159 **160 ± 9.63** (157–163)	−0.25 −0.16 ± 0.83 (−0.49–0.17)
Kruskal–Wallis (*p*)	*p* = 0.108	***p* < 0.001**	***p* < 0.001**
Significant differences between adjacent age groups (*p*)	-	**U11/U12:** ***p* < 0.001**	**U11/U12:** ***p* < 0.001**

**Table 3:** Physical performance for the different age groups. Performance maxima marked in bold.

	Agility T-Half Test (s)	Medicine Ball Throw (m)	Stork Balance Test (s)	Hand Grip Strength (N)
	Median Mean ± SD (95% CI)	Median Mean ± SD (95% CI)	Median Mean ± SD (95% CI)	Median Mean ± SD (95% CI)
U11 (n = 23)	13.1 14.4 ± 2.01 (12.6–14.2)	3.20 3.27 ± 0.79 (2.95–3.60)	2.00 1.81 ± 0.40 (1.64–1.99)	19.0 18.8 ± 3.47 (16.3–21.2)
U12 (n = 23)	13.5 14.6 ± 2.43 (13.8–15.4)	3.50 3.48 ± 0.53 (3.16–3.80)	2.09 **2.09 ± 0.27** (1.91–2.27)	18.5 20.2 ± 5.77 (17.7–22.6)
U13 (n = 23)	12.0 **12.1 ± 1.10** (11.3–12.9)	3.40 **3.56 ± 0.94** (3.24–3.88)	2.00 1.87 ± 0.56 (1.69–2.04)	22.0 **23.3 ± 7.62** (20.9–25.7)
Kruskal–Wallis (*p*)	***p* < 0.001**	*p* = 0.435	*p* = 0.074	***p* = 0.033**
Significant differences between adjacent age groups (*p*)	**U12/U13:** ***p* < 0.001**	-	-	-

**Table 4:** Academic achievement parameters for the different age groups. Performance maxima marked in bold.

	Mathematics	Science	Arabic
	Median Mean ± SD (95% CI)	Median Mean ± SD (95% CI)	Median Mean ± SD (95% CI)
U11 (n = 23)	75.0 79.8 ± 5.74 (76.5–83.0)	85.0 84.8 ± 5.54 (81.8–87.7)	90.0 **88.5 ± 7.45** (84.0–92.0)
U12 (n = 23)	100 **92.8 ± 8.77** (89.6–96.1)	100 **92.8 ± 8.77** (89.9–96.0)	85.0 85.7 ± 9.33 (82.1–89.2)
U13 (n = 23)	85.0 83.4 ± 8.56 (80.4–87.0)	85.0 84.4 ± 6.62 (81.4–87.3)	80.0 79.6 ± 8.52 (76.0–83.1)
Kruskal–Wallis (*p*)	***p* < 0.001**	***p* < 0.001**	***p* = 0.002**
Significant differences between adjacent age groups (*p*)	**U11/U12:** ***p* < 0.001** **U12/U13: ** ***p* < 0.001**	**U11/U12:** ***p* < 0.001** **U12/U13:** ***p* < 0.001**	-

**Table 5:** Physical activity parameters (MET-minutes/week) for the different age groups. Maxima marked in bold.

	Vigorous Physical Activities	Moderate Physical Activities	Walking	All Physical Activities
	Median Mean ± SD (95% CI)	Median Mean ± SD (95% CI)	Median Mean ± SD (95% CI)	Median Mean ± SD (95% CI)
U11 (n = 23)	800 885 ± 723 (599–1172)	480 **456 ± 321** (316–595)	198 340 ± 270 (170–510)	1530 1681 ± 965 (1246–2116)
U12 (n = 23)	720 800 ± 651 (513–1087)	240 423 ± 337 (283–562)	198 481 ± 476 (311–651)	1785 1703 ± 1050 (1268– 2139)
U13 (n = 23)	960 **943 ± 689** (656–1229)	240 369 ± 349 (229–509)	528 **571 ± 449** (401–741)	1866 **1882 ± 1117** (1447–2318)
Kruskal–Wallis (*p*)	*p* = 0.779	*p* = 0.677	*p* = 0.163	*p* = 0.774

No significant differences between adjacent age groups (*p*).

## Data Availability

The datasets generated and/or analyzed during the present study are available from the corresponding author upon reasonable request.

## References

[1] Erlandson M.C., Sherar L.B., Mosewich A.D., Kowalski K.C., Bailey D.A., Baxter-Jones A.D. (2011). Does controlling for biological maturity improve physical activity tracking. Med. Sci. Sports Exerc..

[2] Gebremariam M.K., Bergh I.H., Andersen L.F., Ommundsen Y., Bjelland M., Lien N. (2012). Stability and change in potential correlates of physical activity and association with pubertal status among Norwegian children in the transition between childhood and adolescence. Int. J. Behav. Nutr. Phys. Act..

[3] Silva A.F., Alvurdu S., Akyildiz Z., Badicu G., Greco G., Clemente F.M. (2022). Variations of the Locomotor Profile, Sprinting, Change-of-Direction, and Jumping Performances in Youth Soccer Players: Interactions between Playing Positions and Age-Groups. Int. J. Environ. Res. Public Health.

[4] Williams A.M., Reilly T. (2000). Talent identification and development in soccer. J. Sports Sci..

[5] Leyhr D., Bergmann F., Schreiner R., Mann D., Dugandzic D., Höner O. (2021). Relative Age-Related Biases in Objective and Subjective Assessments of Performance in Talented Youth Soccer Players. Front. Sports Act. Living.

[6] McMorris T., Sproule J., Draper S., Child R. (2000). Performance of a psychomotor skill following rest, exercise at the plasma epinephrine threshold and maximal intensity exercise. Percept. Mot. Skills.

[7] O’Brien-Smith J., Bennett K.J.M., Fransen J., Smith M.R. (2020). Same or different? A comparison of anthropometry, physical fitness and perceptual motor characteristics in male and female youth soccer players. Sci. Med. Footb..

[8] Sørensen A., Haugen E.C., van den Tillaar R. (2022). Is There a Sex Difference in Technical Skills among Youth Soccer Players in Norway?. Sports.

[9] Westerterp K.R. (2013). Physical activity and physical activity induced energy expenditure in humans: Measurement, determinants, and effects. Front. Physiol..

[10] McCarthy C., Warne J.P. (2022). Gender differences in physical activity status and knowledge of Irish University staff and students. Sport Sci. Health.

[11] Penedo F.J., Dahn J.R. (2005). Exercise and well-being: A review of mental and physical health benefits associated with physical activity. Curr. Opin. Psychiatry.

[12] Hardy L.L., Mihrshahi S., Drayton B.A. (2016). Schools BANSW. Physical Activity and Nutrition Survey (SPANS).

[13] Lahti A. (2019). Physical Activity in Childhood and Adolescence. Ph.D. Thesis.

[14] Tremblay M.S., LeBlanc A.G., Kho M.E., Sauners T.J., Larouche R., Colley R.C., Goldfield G., Gorber S.C. (2011). Systematic review of sedentary behaviour and health indicators in school-aged children and youth. Int. J. Behav. Nutr. Phys. Act..

[15] Hermassi S., Bartels T., Hayes L.D., Schwesig R. (2022). Fitness, Fatness, and Academic Attainment in Male Schoolchildren from a Soccer Academy. Int. J. Environ. Res. Public Health.

[16] Carson V., Hunter S., Kuzik N., Gray C.E., Poitras V.J., Chaput J.P., Saunders T.J., Katzmarzyk P.T., Okely A.D., Gorber S.C. (2016). Systematic review of sedentary behaviour and health indicators in school-aged children and youth: An update. Appl. Physiol. Nutr. Metab..

[17] Wu X.Y., Han L.H., Zhang J.H., Luo S., Hu J.W., Sun K. (2017). The influence of physical activity, sedentary behavior on health-related quality of life among the general population of children and adolescents: A systematic review. PLoS ONE.

[18] Malina R.M., Katzmarzyk P.T. (2006). Physical activity and fitness in an international growth standard for preadolescent and adolescent children. Food Nutr. Bull..

[19] Sherar L.B., Cumming S.P., Eisenmann J.C., Baxter-Jones A.D., Malina R.M. (2010). Adolescent biological maturity and physical activity: Biology meets behavior. Pediatr. Exerc. Sci..

[20] Smart J.E., Cumming S.P., Sherar L.B., Standage M., Neville H., Malina R.M. (2012). Maturity associated variance in physical activity and health-related quality of life in adolescent females: A mediated effects model. J. Phys. Act. Health.

[21] Summers-Effler E. (2004). Little girls in women’s bodies: Social interaction and the strategizing of early breast development. Sex Roles.

[22] Wickel E.E., Eisenmann J.C., Welk G.J. (2009). Maturity-related variation in moderate-to-vigorous physical activity among 9–14 year olds. J. Phys. Act. Health.

[23] Rauch F., Bailey D.A., Baxter-Jones A., Mirwald R., Faulkner R. (2004). The ‘muscle-bone unit’ during the pubertal growth spurt. Bone.

[24] Hermassi S., Chelly M.S., Michalsik L.B., Sanal N.E.M., Hayes L.D., Cadenas-Sanchez C. (2021). Relationship between fatness, physical fitness, and academic performance in normal weight and overweight schoolchild handball players in Qatar State. PLoS ONE.

[25] Coster M. (2017). The Effects of Increased Physical Activity on Fracture Risk, Musculoskeletal Development, and Academic Achievement. Ph.D. Thesis.

[26] Mavilidi M.F., Drew R., Morgan P.J., Lubans D.R., Schmidt M., Riley N. (2020). Effects of different types of classroom physical activity breaks on children’s on-task behaviour, academic achievement and cognition. Acta Paediatr..

[27] Troiano R.P., Berrigan D., Dodd K.W., Masse L.C., Tilert T., McDowell M. (2008). Physical activity in the United States measured by accelerometer. Med. Sci. Sports Exerc..

[28] Dumith S.C., Gigante D.P., Domingues M.R., Kohl H.W. (2011). Physical activity change during adolescence: A systematic review and a pooled analysis. Int. J. Epidemiol..

[29] McCormack L.A., Meendering J. (2016). Diet and Physical Activity in Rural vs Urban Children and Adolescents in the United States: A Narrative Review. J. Acad. Nutr. Diet..

[30] Miller J., Pereira M., Wolfson J., Laska M., Nelson T., Neumark-Sztainer D. (2018). Developmental Trends and Determinants of Physical Activity from Adolescence to Adulthood Differ by Ethnicity/Race and Sex. J. Phys. Act. Health.

[31] Farooq A., Martin A., Janssen X., Wilson M.G., Gibson A.-M., Hughes A., Reilly J.J. (2020). Longitudinal changes in moderate-to-vigorous-intensity physical activity in children and adolescents: A systematic review and meta-analysis. Obes. Rev..

[32] Reilly J.J. (2016). When does it all go wrong? Longitudinal studies of changes in moderate-to-vigorous-intensity physical activity across childhood and adolescence. J. Exerc. Sci. Fit..

[33] Hermassi S., Hayes L.D., Schwesig R. (2021). Differences in Fitness and Academic Attainment between Obese, and Non Obese School-Age Adolescent Handball Players: An Explorative, Cross-Sectional Study. Appl. Sci..

[34] Moore S.A., McKay H.A., Macdonald H., Nettlefold L., Baxter-Jones A.D.G., Cameron N., Brasher P.M.A. (2015). Enhancing a somatic maturity prediction model. Med. Sci. Sports Exerc..

[35] Miller D. Measurement by the Physical Educator: Why and How? Brown and Benchmark: Madison, WI, USA, 2002.

[36] Negrete R.J., Hanney W.J., Kolber M.J., Davies G.J., Ansley M.K., McBride A.B., Overstreet A.L. (2010). Reliability, minimal detectable change, and normative values for tests of upper extremity function and power. J. Strength Cond. Res..

[37] Sassi R.H., Dardouri W., Yahmed M.H., Gmada N., Mahfoudhi M.E., Gharbi Z. (2009). Relative and absolute reliability of a modified agility T-test and its relationship with vertical jump and straight sprint. J. Strength Cond. Res..

[38] Hermassi S., Hayes L.D., Bartels T., Schwesig R. (2023). Differences in body composition, static balance, field test performance, and academic achievement in 10-12-year-old soccer players. Front. Physiol..

[39] Hsieh S.S., Tsai J.R., Chang S.H., Ho J.Y., Chen J.F., Chen P.H., Sung Y.T., Hung T.M. (2019). The subject-dependent, cumulative, and recency association of aerobic fitness with academic performance in Taiwanese junior high school students. BMC Pediatr..

[40] Craig C.L., Marshall A.L., Sjöström M., Bauman A.E., Booth M.L., Ainsworth B.E., Pratt M., Ekelund U., Yngve A., Sallis J.F. (2003). International physical activity questionnaire: 12-country reliability and validity. Med. Sci. Sports Exerc..

[41] Hermassi S., Konukman F., Hayes L.D., Schwesig R. (2023). Physical Education and Gender Differences in Physical Activity, Sedentary Behavior Related to Academic Success of Science-Related Courses for Children in the State of Qatar. Appl. Sci..

[42] Kowalski K.C., Crocker R.E., Donen R.M. (2004). The Physical Activity Questionnaire for Older Children (PAC-C) and Adolescents (PAQ-A) Manual.

[43] Wang C., Chen P., Zhuang J. (2013). Validity and reliability of international physical activity questionnaire-short form in Chinese youth. Res. Q. Exerc. Sport.

[44] Bortz J. (1999). Basic Principle of One-Factorial Analysis of Variance. Statistics for Social Scientists.

[45] Richardson J.T.E. (2011). Eta squared and partial eta squared as measures of effect size in educational 416 research. Educ. Res. Rev..

[46] Cohen J. (1988). The Effect Size. Statistical Power Analysis for the Behavioural Sciences.

[47] Willimczik K. (1997). Statistik im Sport: Grundlagen, Verfahren, Anwendungen.

[48] Calsamiglia C., Loviglio A. (2020). Maturity and school outcomes in an inflexible system: Evidence from Catalonia. SERIEs.

[49] Drenowatz C., Grieve G.L., DeMello M.M. (2015). Change in energy expenditure and physical activity in response to aerobic and resistance exercise programs. Springerplus.

[50] Fairclough S.J., Ridgers N.D. (2010). Relationships between maturity status, physical activity, and physical self-perceptions in primary school children. J. Sports Sci..

[51] Reiner M., Niermann C., Jekauc D., Woll A. (2013). Long-term health benefits of physical activity—A systematic review of longitudinal studies. BMC Public Health.

[52] Soini A., Tammelin T., Sääkslahti A., Anthony W., Villberg J., Kettunen T., Mehtälä A., Pskiparta M. (2013). Seasonal and daily variation in physical activity among three-year-old Finnish preschool children. Early Child Dev. Care.

[53] Wyszyńska J., Matłosz P., Szybisty A., Lenik P., Dereń K., Mazur A., Herbert J. (2020). Obesity and Body Composition in Preschool Children with Different Levels of Actigraphy-Derived Physical Activity—A Cross-Sectional Study. J. Clin. Med..

[54] Statler J., Wilk P., Timmons B.W., Colley R., Tucker P. (2020). Habitual physical activity levels and sedentary time of children in different childcare arrangements from a nationally representative sample of Canadian preschoolers. J. Sport Health Sci..

[55] Cooper A.R., Goodman A., Page A.S., Sherar L.B., Esliger D.W., van Sluijs E.M.V., Andersen L.B., Anderssen S., Cardon G., Davey R. (2015). Objectively measured physical activity and sedentary time in youth: The International children’s accelerometry database (ICAD). Int. J. Behav. Nutr. Phys. Act..

[56] Pate R.R., Schenkelberg M.A., Dowda M., McIver K.L. (2019). Group-based physical activity trajectories in children transitioning from elementary to high school. BMC Public Health.

[57] Malina R.M. (2014). Top 10 research questions related to growth and maturation of relevance to physical activity, performance, and fitness. Res. Q. Exerc. Sport..

[58] Francis S.L., Morrissey J.L., Letuchy E.M., Levy S.M., Janz K.F. (2013). Ten-year objective physical activity tracking: Iowa bone development study. Med. Sci. Sports Exerc..

[59] Yim S.Y., Cho J.R., Lee Y. (2003). Normative data and developmental characteristics of hand function for elementary school children in Suwon Area of Korea: Grip, pinch and dexterity study. J. Korean Med. Sci..

[60] Omar M.T., Alghadir A., Al Baker S. (2015). Norms for hand grip strength in children aged 6–12 years in Saudi Arabia. Dev. Neurorehabil..

[61] Hager-Ross C., Rosblad B. (2002). Norms for grip strength in children aged 4–16 years. Acta Paediatr..

[62] Jones M., Hitchen P., Stratton G. (2000). The importance of considering biological maturity when assessing physical fitness measures in girls and boys aged 10 to 16 years. Ann. Hum. Biol..

[63] Al Alwan I., Felimban N., Altwaijri Y., Tamim H., Shoukri M., Tamimi W. (2010). Onset among boys in Riyadh, Saudi Arabia. Clin. Med. Insights Pediatr..

[64] Robertson A., Deitz C.J. (1988). A description of grip strength in preschool children. Am. J. Occup. Ther..

[65] Fullwood D. (1986). Australian norms for hand and finger strength boys and girls aged 5–12 years. Aust. Occup. Ther. J..

[66] Jeune B., Skytthe A., Cournil A., Greco V., Gampe J., Berardelli M., Andersen-Ranberg K., Passarino G., Debenedictis G., Robine J.M. (2006). Handgrip strength among nonagenarians and centenarians in three European regions. J. Gerontol. A Biol. Sci. Med. Sci..

[67] Alotaibi T., Almuhanna R., Alhassan J., Alqadhib E., Mortada E., Alwhaibi R. (2020). The Relationship between Technology Use and Physical Activity among Typically-Developing Children. Healthcare.

[68] Thomas J.R., Gallagher J.D., Purvis G.J. (1981). Reaction time and anticipation time: Effects of development. Res. Q. Exerc. Sport.

[69] Zemkova E., Jelen M., Kovacikova Z., Olle G., Vilman T., Hamar D. (2012). Power outputs in the concentric phase of resistance exercises performed in the interval mode on stable and unstable surfaces. J. Strength Cond. Res..

[70] Dykiert D., Der G., Starr J.M., Deary I.J. (2012). Sex differences in reaction time mean and intraindividual variability across the life span. Dev. Psychol..

[71] Fiorilli G., Iuliano E., Mitrotasios M., Pistone E.M., Aquino G., Calcagno G., di Cagno A. (2017). Are change of direction speed and reactive agility useful for determining the optimal field position for young soccer players?. J. Sports Sci. Med..

[72] Lloyd R., Oliver J., Faigenbaum A., Myer G.D., De Ste Croix M.B.A. (2014). Chronological age vs. biological maturation: Implications for exercise programming in youth. J. Strength Cond. Res..

[73] Hermassi S., Sellami M., Fieseler G., Bouhafs E.G., Hayes L.D., Schwesig R. (2020). Differences in Body Fat, Body Mass Index, and Physical Performance of Specific Field Tests in 10-to-12-Year-Old School-Aged Team Handball Players. Appl. Sci..

[74] Schmid M., Conforto S., Lopez L., D’Alessio T. (2007). Cognitive load affects postural control in children. Exp. Brain Res..

[75] Villarrasa-Sapiña I., Estevan I., Gonzalez L.M., Marco-Ahulló A., García-Massó X. (2019). Dual task cost in balance control and stability in children from 4–7 years old. Early Child Dev. Care.

[76] Ghanbarzadeh A., Azadian E., Majlesi M., Jafarnezhadgero A.A., Akrami M. (2022). Effects of Task Demands on Postural Control in Children of Different Ages: A Cross-Sectional Study. Appl. Sci..

[77] Urruticoechea A., Oliveri A., Vernazza E., Giménez-Dasí M., Martínez-Arias R., Martín-Babarro J. (2021). The Relative Age Effects in Educational Development: A Systematic Review. Int. J. Environ. Res. Public Health.

[78] Aliprantis D. (2014). When Should Children Start School?. J. Hum. Cap..

[79] Dicks A., Lancee B. (2018). Double Disadvantage in School? Children of Immigrants and the Relative Age Effect: A Regression Discontinuity Design Based on the Month of Birth. Eur. Sociol. Rev..

[80] Thoren K., Heinig E., Brunner M. (2016). Relative age effects in mathematics and reading: Investigating the generalizability across students, time and classes. Front. Psychol..

[81] Dhuey E., Figlio D., Karbownik K., Roth J. (2019). School Starting Age and Cognitive Development. J. Policy Anal. Manag..

[82] Cook P., Kang S. (2018). The School-Entry-Age Rule Affects Redshirting Patterns and Resulting Disparities in Achievement.

[83] Diouf A., Thiam M., Idohou-Dossou N., Diongue O., Mégné N., Diallo K., Sembene P.M., Wade S. (2016). Physical Activity Level and Sedentary Behaviors among Public School Children in Dakar (Senegal) Measured by PAQ-C and Accelerometer: Preliminary Results. Int. J. Environ. Res. Public Health.

[84] Díaz-Quesada G., Gálvez-Calabria M.L.Á., Connor J.D., Torres-Luque G. (2022). When Are Children Most Physically Active? An Analysis of Preschool Age Children’s Physical Activity Levels. Children.

